# Associations of Dietary Omega-3 and Omega-6 Fatty Acids, Obesity, and Psychological Stress with Fatigue in Patients with Chronic Obstructive Pulmonary Disease: A Cross-Sectional Study

**DOI:** 10.3390/nu18020355

**Published:** 2026-01-22

**Authors:** Halime Selen, Beste Atabek, Berfin Gegez, Ayşenur Sağ, Burcu Nur Gülbahar, İbrahim Ethem Doğdu, Alperen Aksakal, Metin Akgün

**Affiliations:** 1 Faculty of Health Sciences, Department of Nutrition and Dietetics, Ağrı İbrahim Çeçen University, Ağrı 04100, Türkiye; berfinggz11@hotmail.com; 2 Faculty of Health Sciences, Department of Nutrition and Dietetics, Mardin Artuklu University, Mardin 47100, Türkiye; 3 Department of Pulmonary Medicine, School of Medicine, Ağrı İbrahim Çeçen University, Ağrı 04100, Türkiye; atabekbestee@gmail.com (B.A.); akgunm@gmail.com (M.A.); 4 Department of Pulmonary Medicine, School of Medicine, Atatürk University, Erzurum 25030, Türkiye; sagayse717@gmail.com (A.S.); burcunurt25@gmail.com (B.N.G.); nexon0631@gmail.com (İ.E.D.); alperennaksakal@hotmail.com (A.A.)

**Keywords:** chronic obstructive pulmonary disease, fatigue, obesity, omega-3, omega-6, stress

## Abstract

**Background/Aim**: Fatigue is a common symptom in individuals with chronic obstructive pulmonary disease (COPD) and is associated with reduced quality of life. The aim of this study was to evaluate the relationships between dietary omega-3 (*n*-3) and omega-6 (*n*-6) fatty acid intake, obesity, and stress with fatigue in patients with COPD. **Materials and Methods**: This descriptive cross-sectional study was conducted between 1 February and 31 July 2025, in the pulmonary outpatient clinics of Ağrı Training and Research Hospital in Ağrı and Atatürk University Research Hospital in Erzurum, Türkiye. Study data were collected using a General Information Questionnaire, the COPD and Asthma Fatigue Scale (CAFS), the Perceived Stress Scale (PSS), and an Adult Semi-Quantitative Food Frequency Questionnaire. Higher CAFS scores indicate greater fatigue severity, while higher PSS scores reflect higher perceived stress. **Results**: CAFS scores correlated strongly with perceived stress (r = 0.718, *p* < 0.001) and moderately with COPD exacerbation frequency (r = 0.426, *p* < 0.001). Although higher *n*-3 intake was inversely associated with fatigue in univariate analyses, this association weakened after adjustment, suggesting that fatty acid composition was not an independent determinant of fatigue. The *n*-6/*n*-3 ratio showed a weak positive correlation with fatigue (r = 0.184, *p* = 0.024). Female reported higher fatigue levels than male (mean [SD], 60.2 [19.3] vs. 51.9 [19.8]; *p* = 0.042), and patients with comorbid conditions had higher fatigue scores than those without comorbidities (58.1 [18.3] vs. 46.8 [19.4]; *p* = 0.001). Smoking status was not significantly associated with fatigue (*p* = 0.788). In backward multiple linear regression analysis, perceived stress emerged as the strongest independent predictor of fatigue (β = 0.519, *p* < 0.001). Comorbidity presence (β = 0.206, *p* = 0.030) and smoking status (β = 0.178, *p* = 0.026) were also significant, while exacerbation frequency (*p* = 0.062) and female (*p* = 0.053) showed borderline associations. **Conclusions**: These findings indicate that fatigue in COPD is primarily influenced by psychosocial stress and multimorbidity, highlighting the importance of integrative management approaches that address mental health burden and comorbid conditions alongside respiratory treatment.

## 1. Introduction

Chronic obstructive pulmonary disease (COPD) is a chronic pulmonary disease characterized by persistent airflow limitation and airway remodeling, typically presenting chronic bronchitis and emphysematous changes following long-term exposure to inhaled noxious substances [1]. Exposure to tobacco smoke, including the use of tobacco-related products, continues to represent the predominant etiological factor for COPD on a global scale [2]. Patients with COPD frequently present with multiple coexisting pulmonary and extrapulmonary conditions, including bronchiectasis, asthma, cardiovascular disorders, sleep apnea, nutritional impairment, and skeletal muscle dysfunction, demonstrating the multisystem involvement of the disease [3]. Globally, COPD is among the major contributors to mortality, with over three million deaths reported in 2019 according to the World Health Organization [4].

The development of COPD is widely attributed to prolonged inhalational exposure to noxious agents, which initiates sustained inflammatory processes within the airways and lung parenchyma [1,5]. This inflammation results in progressive lung tissue damage and emphysema, which impairs gas exchange, reduces lung elasticity, and limits airflow, ultimately contributing to decreased lung function [6,7].

Fatigue is a commonly experienced symptom among patients with COPD and reflects a multisystem feature associated with marked impairment in quality of life. Reported prevalence estimates range from 17% to 95%, depending on study populations and assessment methods [8]. Recent evidence indicates that the global prevalence of fatigue among individuals with COPD is approximately 59%, although notable regional variation exists [9]. Fatigue in COPD has a multifactorial etiology and is closely associated with both non-modifiable factors, such as age, sex, and genetic characteristics, and modifiable factors, including tobacco exposure, exacerbation frequency, disease duration and age at diagnosis, symptom burden, air pollution, physical activity, sleep patterns, nutritional status, obesity or undernutrition, comorbidities, psychological status, education level, and socioeconomic conditions [10]. Recognition of modifiable risk factors is therefore essential for improving symptom control and disease management.

An expanding body of evidence has examined how polyunsaturated fatty acids—particularly omega-3 (*n*-3) and omega-6 (*n*-6) fatty acids—may influence inflammatory mechanisms, oxidative balance, and metabolic regulation. Accordingly, the present study sought to examine the relationships between dietary intake of omega-3 fatty acids—α-linolenic acid (ALA), eicosapentaenoic acid (EPA), and docosahexaenoic acid (DHA)—and omega-6 fatty acids—linoleic acid (LA) and arachidonic acid (AA)—together with obesity and perceived stress, in relation to fatigue severity among patients with COPD. The analysis also accounted for the potential contributions of comorbid conditions, smoking status, exacerbation frequency, and sex. Unlike previous studies that have examined these factors separately, this study uniquely integrates dietary, physiological, and psychosocial variables to provide a comprehensive understanding of fatigue determinants, highlighting potential targets for more effective fatigue management in COPD.

## 2. Materials and Methods

### 2.1. Study Period, Design, and Setting

This cross-sectional study was conducted from 1 February to 31 July 2025. Data were collected in the pulmonary outpatient clinics of Ağrı Training and Research Hospital in Ağrı and Atatürk University Research Hospital in Erzurum, Türkiye. Written and verbal informed consent was obtained from all participants before the enrollment.

### 2.2. Study Population and Sample

A total of 150 patients with stable COPD were included in this cross-sectional study.

The study population consisted of patients with stable chronic obstructive pulmonary disease attending the pulmonary outpatient clinics during the study period. COPD was diagnosed according to GOLD criteria based on post-bronchodilator spirometry demonstrating an FEV_1_/FVC ratio < 0.70 [1]. Participants were enrolled consecutively using a convenience sampling approach.

The sample size was estimated to enable assessment of the association between dietary unsaturated fatty acid intake and fatigue as a continuous outcome in patients with COPD using a multivariable linear regression approach. Fatigue was assessed using the COPD and Asthma Fatigue Scale (CAFS). Assuming a moderate effect size for a continuous outcome and adjustment for age and sex, a minimum sample size of 120 participants was estimated to achieve 80% power at a two-sided alpha level of 0.05. A total of 150 patients were ultimately included in the study.

### 2.3. Inclusion Criteria

Participants were included if they satisfied all the following criteria:

(a) Individuals aged 40 years or older.
(b) Clinically stable COPD at the time of hospital evaluation, defined as no acute exacerbation or need for systemic corticosteroids or antibiotics within the preceding four weeks [1].
(c) No use of any dietary supplements, particularly those containing omega-3 or omega-6 fatty acids (e.g., fish oil, krill oil, flaxseed oil, evening primrose oil).
(d) Ability to communicate independently with preserved cognitive function.
(e) Obtaining informed consent to partake in the study.

### 2.4. Exclusion Criteria

Participants were excluded only if they were unable to complete the questionnaires reliably during the study procedures or had missing clinical, dietary, or questionnaire data.

### 2.5. Data Collection Instruments

The General Information Questionnaire, the COPD and Asthma Fatigue Scale (CAFS), and the Perceived Stress Scale (PSS) were used to gather study data. The Adult Semi-Quantitative Food Frequency Questionnaire was used to measure the participants’ intake of *n*-3 and *n*-6 fatty acids.

#### 2.5.1. General Information Questionnaire

The General Information Questionnaire was used to collect data on age, sex, body weight (kg), height (m), smoking status (pack-years; categorized as never, former, or current smoker), age at COPD diagnosis, presence of comorbid conditions, COPD stage, pulmonary function test results (FEV_1_ and FVC), and the number of hospital visits due to COPD exacerbations during the preceding year. Comorbid conditions were documented based on patient self-report and review of medical records, and COPD stage was classified according to the GOLD framework based on dyspnea severity and history of exacerbations [1]. Anthropometric data were collected using standardized procedures, with standing height measured to the nearest 0.1 cm by a digital stadiometer and body weight recorded to the nearest 0.1 kg using calibrated scales. Body mass relative to stature was expressed as kilograms per square meter (kg/m^2^). In accordance with World Health Organization reference ranges, values below 18.5 kg/m^2^ were classified as underweight, those between 18.5 and 24.9 kg/m^2^ as normal weight, between 25.0 and 29.9 kg/m^2^ as overweight, and values of 30.0 kg/m^2^ or higher as obese [11].

#### 2.5.2. COPD and Asthma Fatigue Scale (CAFS)

Fatigue was assessed using the COPD and Asthma Fatigue Scale (CAFS), developed by Revicki et al. (2010) and validated for the Turkish population by Arslan and Öztunç (2013) [12,13]. The scale consists of 12 items; each rated on a five-point Likert scale ranging from “never (1)” to “very often (5)”. Item scores were summed to obtain a total raw score ranging from 12 to 60, with higher scores indicating greater fatigue severity.

To facilitate interpretation, raw scores were linearly transformed to a standardized 0–100 scale using the following formula:

$$\mathrm{CAFS}\;\mathrm{score}=\left(\frac{\mathrm{Raw}\;\mathrm{score}-12}{48}\right)\times 100$$

where 12 represents the minimum possible score and 48 represents the possible score range. Higher transformed scores indicate higher levels of fatigue [13].

#### 2.5.3. Perceived Stress Scale (PSS)

Perceived stress was measured using the 14-item Perceived Stress Scale (PSS), originally developed by Cohen et al. (1983) and validated in Turkish by Eskin et al. (2013) [14,15]. Each item is rated on a five-point Likert scale ranging from “never (0)” to “very often (4)”. Items 4, 5, 6, 7, 9, 10, and 13 were reverse-scored prior to analysis. After reverse scoring, all item scores were summed to obtain a total score ranging from 0 to 56. Higher total scores indicate higher perceived stress [15].

#### 2.5.4. Adult Semi-Quantitative Food Frequency Questionnaire

Dietary intake was evaluated using an adult semi-quantitative food frequency questionnaire specifically developed for the Turkish population [16]. Portion sizes were approximated with reference to a standardized food and nutrition photograph atlas [17]. Questionnaire data were subsequently processed using the Nutrition Information System (BeBiS) to estimate individual daily intakes of total omega-3 and omega-6 fatty acids (g/day) [18]. Dietary sources of omega-3 and omega-6 fatty acids were determined based on all food items captured by the food frequency questionnaire. Omega-3 intake was primarily contributed by fish and seafood, nuts and seeds, and selected vegetables, whereas omega-6 intake was mainly derived from vegetable oils, nuts and seeds, cereals, and processed foods containing these oils.

### 2.6. Ethical Approval

Ethical approval was granted by the Scientific Research Ethics Committee of Ağrı İbrahim Çeçen University (approval date: 26 December 2024; approval number: 472). All study procedures were performed in accordance with the principles outlined in the Declaration of Helsinki.

### 2.7. Statistical Analysis

We performed analyses using IBM SPSS Statistics (version 29.0; IBM Corp., Armonk, NY, USA). Continuous variables were summarized as mean (SD) or median (IQR), and categorical variables as counts and percentages. Group comparisons used parametric or nonparametric tests as appropriate. Associations between continuous variables were assessed with Pearson correlation, and independent predictors of fatigue (CAFS score) were identified using backward multiple linear regression. Regression coefficients are reported with corresponding 95% confidence intervals. Variables entered the regression model were selected a priori based on clinical relevance and evidence from previous literature and were retained in the final model if they showed statistical significance. Multicollinearity among variables included in the regression model was assessed using variance inflation factors (VIF), and no evidence of problematic multicollinearity was observed. Model assumptions, including linearity, normality of residuals, and homoscedasticity, were assessed and found to be acceptable. Variables meeting a significance threshold of *p* < 0.05 were retained in the final model. All analyses were two-tailed, with statistical significance set at *p* < 0.05.

## 3. Results

### 3.1. Baseline Characteristics

A total of 150 patients with COPD were included (mean [SD] age 65.19 [9.88] years; 120 [80.0%] male). The mean (SD) body-mass index (BMI) was 28.00 (5.60) kg/m^2^. Twenty (13.3%) participants had never smoked, 87 (58.0%) were former, and 43 (28.7%) were current smokers; the median (IQR) smoking status was 30.00 (15.00–50.00) pack-years. Median (IQR) COPD duration was 4.00 (1.00–7.00) years, and exacerbation frequency 1.00 (0.00–4.00). Spirometry data were available for 127 (84.67%) participants. Apart from spirometry, data for other clinical, dietary, and questionnaire variables were complete and did not require imputation. Mean (SD) FEV_1_ was 1.60 (0.60) L (61.30 [19.30]% predicted), and mean (SD) FVC was 2.80 (1.00) L (85.40 [19.80]% predicted). Comorbidities were present in 90 (60.0%) patients—most commonly hypertension (29.3%) and diabetes (19.3%). Median (IQR) CAFS and PSS scores were 58.00 (40.00–69.00) and 28.00 (20.00–34.00), respectively. The median (IQR) daily intakes were 1.10 (0.90–1.60) g for total *n*-3, 22.30 (11.30–30.80) g for total *n*-6, and 14.60 (6.10–29.10) for the *n*-6/*n*-3 ratio (Table 1).

### 3.2. Correlation Analysis

CAFS correlated strongly with perceived stress (*r* = 0.718, *p* < 0.001) and moderately with COPD exacerbation frequency (*r* = 0.426, *p* < 0.001). This finding indicates a strong association of clinically meaningful magnitude, suggesting that higher perceived stress is closely linked to greater fatigue severity in patients with COPD. Although higher *n*-3 intake was inversely correlated with fatigue in univariate analyses, this nutritional association was attenuated after adjustment, indicating that fatty-acid composition was not an independent determinant of fatigue. The *n*-6/*n*-3 ratio correlated weakly and positively (*r* = 0.184, *p* = 0.024). Female reported greater fatigue than male (mean [SD], 60.2 [19.3] vs. 51.9 [19.8]; *p* = 0.042). Comorbid patients also reported higher fatigue (58.1 [18.3] vs. 46.8 [19.4]; *p* = 0.001). Smoking status was not significantly associated with fatigue (*p* = 0.788) (Table 2). CAFS scores stratified by sex and comorbidity status are presented as median (IQR) in Supplementary Table S1.

### 3.3. Multivariable Analysis

In backward multiple linear regression (Table 3), perceived stress (PSS) emerged as the dominant independent predictor of fatigue (*β* = 0.519, *p* < 0.001). Comorbidity presence (*β* = 0.206, *p* = 0.030) and smoking status (*β* = 0.178, *p* = 0.026) were also significant. This finding indicates that the association between smoking status and fatigue became apparent after adjustment for potential confounders included in the multivariable model. Exacerbation frequency (*p* = 0.062) and female (*p* = 0.053) showed borderline effects. Nutritional variables (*n*-3, *n*-6, and the *n*-6/*n*-3 ratio) and pulmonary indices (FEV_1_, FVC) were excluded from the model because they were not independently associated with fatigue. The final model explained 59 % of the variance in fatigue (adjusted R^2^ = 0.59) (Table 3).

## 4. Discussion

In this cross-sectional study of patients with COPD, perceived psychological stress emerged as the strongest independent determinant of fatigue severity. Comorbid conditions and smoking behavior were also independently associated with higher fatigue levels, whereas exacerbation frequency and female showed borderline associations. Although dietary *n*-3 and *n*-6 fatty acid intakes were related to fatigue in univariate analyses, these associations were attenuated after multivariable adjustment, suggesting that dietary fatty acid composition may not independently influence fatigue once psychosocial and clinical factors are accounted for. Importantly, due to the cross-sectional design, causal inferences cannot be drawn, and the observed associations should be interpreted as correlational rather than directional.

### 4.1. Psychological Stress and Fatigue

Our findings align with previous literature demonstrating strong links between psychological stress, anxiety, depression, and fatigue in individuals with COPD. Offor et al. (2025) reported that higher perceived stress scores were associated with poorer respiratory health, reduced quality of life, and elevated oxidative stress markers [19]. Homętowska et al. (2022) found that elevated depression and anxiety were associated with greater fatigue, and Kazak Salti et al. (2025) highlighted stress as the most commonly reported cause of fatigue in patients with COPD [20,21]. These studies, together with our findings, underscore the importance of addressing mental health in the comprehensive management of fatigue in COPD. These results suggest that integrative interventions targeting both physical and psychosocial domains may be particularly beneficial. For example, combining pulmonary rehabilitation programs with stress-management strategies—such as cognitive-behavioral therapy, mindfulness-based interventions, or relaxation techniques—could potentially reduce fatigue and improve overall quality of life in COPD patients.

### 4.2. Comorbid Conditions

Comorbidity burden was another significant predictor of fatigue in our study. Prior research indicates that patients with COPD and additional chronic diseases have a markedly increased likelihood of severe fatigue [8,22]. In Türkiye, overweight and obesity were linked to higher fatigue levels among older adults with COPD [23]. These observations reinforce the need for a holistic approach to fatigue management that considers comorbidities alongside pulmonary function.

### 4.3. Smoking Status

Smoking remains a major contributor to COPD pathogenesis and disease progression [24]. Our results show that current and former smoking behaviors are independently associated with fatigue, likely reflecting their cumulative impact on systemic inflammation, comorbidity burden, and oxidative stress [25,26]. While this section is extensive in prior literature, in our study, smoking acted as a secondary predictor of fatigue, highlighting the need for continued emphasis on smoking cessation as part of overall COPD management.

### 4.4. Exacerbation Frequency and Sex Differences

Although exacerbation frequency was only borderline associated with fatigue, prior studies suggest that frequent exacerbations worsen pulmonary function and quality of life [27,28,29]. Our study also found that females reported higher fatigue levels than males; however, evidence on sex differences in COPD-related fatigue remains inconsistent [23,30,31]. These discrepancies likely reflect heterogeneity in demographic characteristics, disease severity, and assessment tools.

### 4.5. Dietary Polyunsaturated Fatty Acids (PUFAs)

Although univariate analyses suggested an association between dietary *n*-3 and *n*-6 fatty acids and fatigue, these relationships were attenuated after adjustment for psychosocial and clinical factors. This finding indicates that the effects of dietary fatty acids on fatigue may be partially mediated or confounded by stress, comorbidity, and obesity. Previous studies have shown mixed results regarding PUFAs in COPD, with some reporting benefits of higher *n*-3 intake for symptom reduction and quality of life [32,33], whereas others found no significant associations [34]. Mechanistically, *n*-3 fatty acids may reduce systemic inflammation through anti-inflammatory eicosanoid production and NF-κB inhibition [35], whereas excessive *n*-6 intake may promote inflammation [36,37]. Our findings suggest that, within this multifactorial context, dietary fatty acids alone do not appear to be primary drivers of fatigue.

### 4.6. Inflammation, Obesity, and Psychosocial Stress

Fatigue in COPD is multifactorial, with contributions from inflammation, oxidative stress, psychological stress, obesity, and nutritional status [38]. Chronic stress can increase cortisol levels and systemic inflammation [39], while adipose-derived proinflammatory cytokines such as TNF-α, IL-1, and IL-6 may further exacerbate fatigue in obese individuals [40]. This context may partly explain why the associations between PUFAs and fatigue were attenuated after multivariable adjustment.

### 4.7. Study Limitations and Strengths

This study has several important limitations that should be considered when interpreting the findings. First, the cross-sectional design precludes any causal inference; therefore, the observed associations should not be interpreted as directional or explanatory relationships. Second, the relatively small sample size and the recruitment of participants from only two centers may limit the generalizability of the results.

In addition, dietary fatty acid intake was assessed using self-reported methods, which are subject to recall bias and measurement error. The absence of objective biomarkers (e.g., plasma fatty acid profiles) of fatty acid status and longitudinal data further necessitates cautious interpretation; thus, the observed associations should be regarded as exploratory rather than confirmatory.

Moreover, several factors closely related to fatigue in COPD—including depression, anxiety, sleep quality, physical activity levels, and other psychosocial or environmental variables—were not assessed and may have contributed to residual confounding.

Despite these limitations, the study offers valuable insights into fatigue in COPD by adopting a multifactorial perspective that integrates dietary, anthropometric, and psychological stress-related factors. To our knowledge, this study represents one of the most comprehensive evaluations of fatigue among individuals with COPD in the Turkish population and underscores the potential importance of integrative and holistic approaches in the management of COPD-related fatigue.

### 4.8. Clinical Implications and Future Directions

Our study highlights the complex interplay of psychological, clinical, and lifestyle factors in COPD-related fatigue. Clinically, these findings suggest that fatigue management should extend beyond pulmonary-focused interventions to include mental health support, comorbidity management, and lifestyle modification. From a research perspective, longitudinal and interventional studies incorporating objective nutritional biomarkers, such as plasma or erythrocyte EPA and DHA levels, are needed to clarify causal relationships and the potential role of dietary polyunsaturated fatty acids in mitigating fatigue. Alternative analytical approaches, including mediation or interaction analyses, may further help to capture the complex relationships between dietary factors, psychological stress, and fatigue. In addition, future work should consider whether the effects of dietary fatty acids differ across specific subgroups, such as GOLD stage, obesity status, or inflammatory profile, and whether stratified analyses may help elucidate potential heterogeneity in these associations, while also integrating other psychological variables, including depression and anxiety. More detailed stratification of comorbid conditions (e.g., cardiovascular, metabolic, or sleep-related disorders) may also help identify which comorbidity profiles most strongly contribute to fatigue in COPD.

## 5. Conclusions

Perceived psychological stress emerged as the strongest independent determinant of fatigue severity in individuals with COPD, while comorbid conditions and smoking were also associated. Exacerbation frequency and female showed borderline associations. Although univariate analyses suggested links between dietary *n*-3 and *n*-6 fatty acid intake and fatigue, these were not independent in multivariable models. These findings highlight that fatigue in COPD is driven primarily by psychosocial and comorbid factors, emphasizing the need for integrative management approaches that include mental health support, smoking cessation, and tailored care for comorbidities alongside respiratory treatment. Given the cross-sectional design, causality cannot be inferred, and longitudinal studies are needed to confirm these relationships. Future research should explore whether targeted interventions to reduce stress and optimize comorbidity management can alleviate fatigue in this population.

## Figures and Tables

**Table 1:** Baseline characteristics of the study population.

Characteristics	Overall (n = 150)
*Demographics*	
*Sex*	
Female, n (%)	30 (20.00)
Male, n (%)	120 (80.00)
Age, years, mean (SD)	65.19 (9.88)
Body mass index, kg/m^2^, mean (SD)	28.00 (5.60)
*BMI categories*	
Underweight (<18.5), n (%)	1 (0.67)
Normal (18.5–24.9), n (%)	51 (34.00)
Overweight (25.0–29.9), n (%)	53 (35.33)
Obese (≥30.0), n (%)	45 (30.00)
*Smoking status*	
Never, n (%)	20 (13.33)
Former, n (%)	87 (58.00)
Current, n (%)	43 (28.67)
Smoking pack-years, median (IQR)	30.00 (15.00–50.00)
*COPD characteristics*	
COPD duration, years, median (IQR)	4.00 (1.00–7.00)
Exacerbations in the past 12 months, median (IQR)	1.00 (0.00–4.00)
GOLD group A, n (%)	42 (28.00)
GOLD group B, n (%)	33 (22.00)
GOLD group E, n (%)	75 (50.00)
*Pulmonary function tests*	
Spirometry available, n (%)	127 (84.67)
FEV_1_, mL, mean (SD)	1.60 (0.60)
FEV_1_, % predicted, mean (SD)	61.30 (19.30)
FVC, mL, mean (SD)	2.80 (1.00)
FVC, % predicted, mean (SD)	85.40 (19.80)
FEV_1_/FVC, mean (SD)	59.30 (9.60)
*Comorbidities*	
Any comorbidity, n (%)	90 (60.00)
Diabetes mellitus, n (%)	29 (19.33)
Hypertension, n (%)	44 (29.33)
Heart failure, n (%)	6 (4.00)
Coronary artery disease, n (%)	1 (0.67)
Valvular heart disease, n (%)	1 (0.67)
*Dietary fatty acids*	
Total *n*-3 intake, g/day, median (IQR)	1.10 (0.90–1.60)
Total *n*-6 intake, g/day, median (IQR)	22.30 (11.30–30.80)
*n*-6/*n*-3 ratio, median (IQR)	14.60 (6.10–29.10)
*n*-*3 fatty acid subcomponents*	
ALA, g/day, median (IQR)	1.00 (0.80–1.40)
EPA, g/day, median (IQR)	0.00 (0.00–0.10)
DHA, g/day, median (IQR)	0.10 (0.00–0.10)
EPA + DHA, g/day, median (IQR)	0.10 (0.10–0.20)
*n*-*6 fatty acid subcomponents*	
LA, g/day, median (IQR)	22.20 (11.20–30.60)
AA, g/day, median (IQR)	0.20 (0.10–0.20)

*Notes:* Data are expressed as mean (SD) for variables with a normal distribution and as median (IQR) for variables with a non-normal distribution, unless stated otherwise. *Abbreviations:* AA, arachidonic acid; ALA, α-linolenic acid; BMI, body mass index; COPD, chronic obstructive pulmonary disease; DHA, docosahexaenoic acid; EPA, eicosapentaenoic acid; FEV_1_, forced expiratory volume in 1 s; FVC, forced vital capacity; LA, linoleic acid; *n*-3, omega-3; *n*-6, omega-6.

**Table 2:** Pearson correlation coefficients between fatigue (CAFS score) and clinical, nutritional, and pulmonary variables in patients with COPD (n = 150).

Variable	r (Pearson)	*p* Value
PSS	0.718	<0.001
COPD exacerbations (past 12 months)	0.426	<0.001
EPA, g/day	−0.404	<0.001
DHA, g/day	−0.426	<0.001
EPA + DHA, g/day	−0.421	<0.001
Total *n*-3 intake, g/day	−0.372	<0.001
ALA, g/day	−0.308	<0.001
LA, g/day	−0.227	0.005
AA, g/day	−0.161	0.049
Total *n*-6 intake, g/day	−0.230	0.005
*n*-6/*n*-3 ratio	0.184	0.024
Age, years	−0.084	0.307
BMI, kg/m^2^	0.081	0.325
Smoking status, pack-years	0.139	0.090
COPD duration, years	0.146	0.074
FEV_1_, mL	−0.172	0.055
FEV_1_, % predicted	−0.058	0.518
FVC, mL	−0.232	0.009
FVC, % predicted	−0.117	0.192
FEV_1_/FVC ratio	−0.065	0.470

*Notes:* Associations between continuous variables were examined using Pearson correlation analysis. Statistical significance was defined as *p* < 0.05. *Abbreviations:* AA, arachidonic acid; ALA, α-linolenic acid; BMI, body mass index; CAFS, COPD and Asthma Fatigue Scale; COPD, chronic obstructive pulmonary disease; DHA, docosahexaenoic acid; EPA, eicosapentaenoic acid; FEV_1_, forced expiratory volume in 1 s; FVC, forced vital capacity; PSS, Perceived Stress Scale.

**Table 3:** Backward multiple linear regression analysis of factors associated with fatigue (CAFS score) in patients with COPD (n = 150).

Predictor	Standardized β	t Value	*p* Value
PSS	0.519	7.46	<0.001
Comorbidity (present)	0.206	2.20	0.030
Smoking status (current/former vs. never)	0.178	2.27	0.026
COPD exacerbations (past 12 months)	0.146	1.89	0.062
Sex (female)	0.142	1.96	0.053
Age (years)	−0.084	−1.50	0.137
BMI (kg/m^2^)	0.082	1.36	0.178
FEV_1_ (% predicted)	0.010	0.34	0.734
FVC (% predicted)	−0.017	−0.29	0.773

*Model characteristics:* Backward elimination multiple linear regression analysis was performed, yielding an adjusted R^2^ of 0.59. Dietary fatty-acid variables, including total *n*-3 intake, total *n*-6 intake, and the *n*-6/*n*-3 ratio, as well as COPD stage according to the GOLD classification, were sequentially removed from the model because they did not show an independent association with fatigue severity (all *p* > 0.30). *Abbreviations:* CAFS, COPD and Asthma Fatigue Scale; COPD, chronic obstructive pulmonary disease; PSS, Perceived Stress Scale; BMI, body mass index; FEV_1_, forced expiratory volume in 1 s; FVC, forced vital capacity.

## Data Availability

The original contributions presented in this study are included in the article/Supplementary Material. Further inquiries can be directed to the corresponding author.

## Supplementary Materials

The following supporting information can be downloaded at: https://www.mdpi.com/article/10.3390/nu18020355/s1, Table S1: CAFS scores stratified by sex and comorbidity status.
