# Supplementary material for: Associations of Dietary Omega-3 and Omega-6 Fatty Acids, Obesity, and Psychological Stress with Fatigue in Patients with Chronic Obstructive Pulmonary Disease: A Cross-Sectional Study
Source: Nutrients. 2026 Jan 22;18(2):355. doi: 10.3390/nu18020355 (PMC12844984; doi:10.3390/nu18020355)
Supplement: Supplementary file 1 [file nutrients-18-00355-s001.zip › nutrients-4102689-SI.pdf]

Supplementary Table S1. CAFS scores stratified by sex and comorbidity status

Sex	Comorbidity status	n	CAFS score, median (IQR)
Male	Absent	45	45.8 (29.2–60.4)
Male	Present	75	62.5 (44.8–70.8)
Female	Absent	13	64.6 (45.8–70.8)
Female	Present	17	64.6 (52.1–75.0)

Abbreviations: CAFS, COPD and Asthma Fatigue Scale; IQR, interquartile range.
